# Fibroblast growth factor 21 inversely correlates with survival in elderly population – the results of the Polsenior2 study

**DOI:** 10.18632/aging.206114

**Published:** 2024-09-18

**Authors:** Gabriela Handzlik, Aleksander J. Owczarek, Andrzej Więcek, Małgorzata Mossakowska, Tomasz Zdrojewski, Anna Chudek, Magdalena Olszanecka-Glinianowicz, Jerzy Chudek

**Affiliations:** 1 Department of Internal Medicine and Oncological Chemotherapy, Faculty of Medical Sciences in Katowice, Medical University of Silesia, Katowice, Poland; 2 Health Promotion and Obesity Management Unit, Department of Pathophysiology, Faculty of Medical Sciences in Katowice, Medical University of Silesia, Katowice, Poland; 3 Department of Nephrology, Transplantation and Internal Medicine, Medical University of Silesia, Katowice, Poland; 4 Study on Aging and Longevity, International Institute of Molecular and Cell Biology, Warsaw, Poland; 5 Division of Preventive Medicine and Education, Medical University of Gdansk, Gdansk, Poland

**Keywords:** fibroblast growth factor 21, aging, survival, population-based study, longevity

## Abstract

Fibroblast growth factor 21 (FGF21) is a liver-secreted hormone involved in the regulation of lipid, glucose, and energy metabolism. Its serum concentration increases with age but also is higher in numerous diseases. FGF21 is being investigated for biomarker properties and as a potential therapeutic target. The present study aimed to assess the prognostic value of FGF21 in an older population-based cohort, the PolSenior2 study participants. In the sub-analysis of 3512 individuals, aged 60 and older, stratified according to FGF21 into tertiles, the survival estimate was worse in participants with middle and high levels compared to the lowest tertile. These results were consistent with univariable Cox regression analysis, in which participants in the middle and the high FGF21 tertiles after adjustment for age had 1.43-fold (HR, 1.31; 95% CI, 1.05 – 1.62) and 2.56-fold (HR, 1.94; 95% CI, 1.59 – 2.37) higher risk for mortality, respectively, compared with those in the lowest tertile. In multivariable Cox regression analysis, the highest levels of FGF21 were associated with increased mortality (HR 1.53; 95% CI, 1.22 – 1.92) independently of co-morbidities and blood parameters. These results indicate that higher serum FGF21 concentration is an independent predictor of all-cause mortality in the general population of older adults.

## INTRODUCTION

Fibroblast growth factor 21 (FGF21) is a hormone secreted mostly by the liver. In smaller quantities is produced by adipose and vascular tissues, skeletal muscles, pancreas, as well as in the brain, exerting differential actions via auto-, para- and endocrine manner. FGF21 effect on target tissues is related to binding to the FGF receptor and co-receptor protein β-klotho. The primary role of FGF21 is the regulation of glucose homeostasis and lipid metabolism [1, 2]. Research on pharmacological treatment with FGF21 analogs and FGF21-mimetic monoclonal antibodies indicates beneficial properties of FGF21 on weight loss, cholesterol and triglyceride level, and serum glucose concentration (via enhancement of insulin sensitivity) [3, 4]. Though, its action exceeds substantially beyond these functions and is considered to be involved, among others, in energy homeostasis and nutrient metabolism, atherosclerosis prevention, and stress responses, as such may become a potential target for the treatment of metabolic disturbances [5].

Experimental findings suggest that higher concentrations of FGF21 attenuate acute metabolic disorders, contributing to extended lifespan [6, 7]. On the other hand, long-lasting elevation of FGF21 is interpreted to be associated with precocious aging and premature death in mice [8]. The effects of FGF21 on target tissues are modulated by a variety of metabolic stimuli and so far, the clinical significance of FGF21 is not entirely elucidated and in some aspects difficult to interpret. However, it does not exclude the role of FGF21 as a potential predictive biomarker, particularly in older populations, in which serum FGF21 concentration is elevated [9]. Incomplete data on the factors regulating FGF21 levels in older adults justifies conducting further research in this field. The present analysis was a sub-study of a large, nationwide, multicentre project and it aimed to determine the relationship between FGF21 serum levels and survival rates in subjects aged 60 and above, a representative of the Polish older adult population.

## RESULTS

### Characteristics of the study population

Serum FGF21 concentration was measured in 3512 subjects [1737 men (49.5%) and 1775 women (50.5%)].

Study group was stratified by gender and according to tertiles of the FGF21 serum concentration and showed variables according to these strata: low [< 177.8 pg/mL for men; < 201.4 pg/mL for women], middle [177.8 – 365.5 pg/mL for men; 201.4 – 409.4 pg/mL for women], and high [> 365.5 pg/mL for men; > 409.4 pg/mL for women;]. The median FGF21 in all tertiles was higher for women than for men. The greatest difference was noted in the lower quartiles (by 20%).

Characteristics of the study subgroups with the values of selected variables according to the FGF21 category are presented in Table 1 for men and Table 2 for women. Participants with middle and high serum concentrations of FGF21 were older compared to those with lower levels of FGF21. Furthermore, they more frequently had hypertension, obesity, diabetes, hyperuricemia, hypercholesterolemia, and hypertriglyceridemia. Female patients with middle and high FGF21 concentration had also significantly increased prevalence of stroke incidents.

**Table 1:** Baseline clinical characteristics of the male participants according to FGF-21 categories (N = 1737; 49.5% of the analyzed cohort).

	**FGF-21 categories**			
	**LOW [N = 579]**	**MIDDLE [N = 579]**	**HIGH [N = 579]**	***p-value***
FGF-21 (pg/mL)	106.3 (69.7 – 141.4)	262.4 (219.3 – 304.8)	583.2 (448.7 – 924.6)	–
Age (years)	74 ± 9	75 ± 10^*^	76 ± 10^#^	< 0.001
Age ≥ 80 years, N (%)	156 (26.9)	182 (31.4)	228 (39.4)^#^	< 0.001
BMI (kg/m^2^)	27.7 ± 4.5	28.1 ± 4.5	28.6 ± 4.6^**^	< 0.01
Obesity, N (%)	164 (28.6)	177 (31.2)	202 (36.1)^**^	< 0.05
Visceral obesity, N (%)	418 (74.3)	452 (80.9)	464 (83.3)	< 0.001
Arterial hypertension, N (%)	428 (73.9)	436 (75.3)	465 (80.6)^**^	< 0.05
Coronary artery disease, N (%)	65 (36.5)	68 (38.2)	67 (34.0)	0.70
Past stroke, N (%)	49 (8.5)	63 (10.9)	64 (11.1)	0.26
Heart failure, N (%)	113 (20.7)	122 (22.3)	136 (24.8)	0.26
Diabetes mellitus, N (%)	134 (23.1)	170 (29.4)^*^	193 (33.3)^#^	< 0.001
Impaired fasting glucose, N (%)	245 (42.3)	273 (47.2)	303 (52.3)^#^	< 0.01
Fasting glucose (mg/dL)	97 (89 – 107)	98 (91 – 114)^**^	101 (92 – 116)^#^	< 0.01
HbA1c (mmol/l)	36 (33 – 40)	37 (34 – 42)^**^	38 (34 – 43)^#^	< 0.001
HOMA-IR	1.78 (1.10 – 2.71)	2.16^#^ (1.35 – 3.54)	2.20^#^ (1.30 – 3.76)	< 0.001
HOMA-IR ≥ 2.0	241 (41.7)	313 (54.3)^#^	314 (54.7)^#^	< 0.001
Hypercholesterolemia, N (%)	404 (69.8)	433 (74.8)	387 (66.8)	< 0.05
Total cholesterol (mg/dL)	177.4 ± 42.3	178.2 ± 44.2	173.8 ± 42.5	0.19
HDL cholesterol (mg/dL)	51.3 ± 13.7	48.6 ± 12.2^**^	47.0 ± 13.8^#^	< 0.001
LDL cholesterol (mg/dL)	111.8 ± 39.4	114.4 ± 40.5	122.7 ± 44.2	0.08
Hypertriglyceridemia, N (%)	85 (14.7)	159 (27.5)^#^	201 (34.7)^#^	< 0.001
Triglycerides (mg/dL)	101 (80 – 130)	113 (87 – 156)^#^	124 (94 – 170)^#^	< 0.001
hs-CRP (mg/L)	1.44 (0.75 – 3.34)	1.87^*^ (0.90 – 3.97)	2.94^#^ (1.36 – 6.39)	< 0.001
hs-CRP > 3 mg/L, N (%)	159 (27.5)	194 (33.5)^*^	283 (48.9)^#^	< 0.001
hs-CRP > 5 mg/L, N (%)	95 (16.4)	110 (19.0)	176 (30.4)^#^	< 0.001
Hyperuricemia, N (%)	116 (20.0)	176 (30.4)^#^	219 (37.8)^#^	< 0.001
Uric acid (mg/dl)	5.82 ± 1.25	6.24 ± 1.44^#^	6.52 ± 1.62^#^	< 0.001
Statins, N (%)	234 (40.4)	244 (42.1)	219 (37.8)	0.32
Fibrates, N (%)	1 (0.2)	5 (0.9)	32 (5.5)^#^	< 0.001
Albumin (g/L)	43.6 (41.8 – 45.1)	43.3 (41.5 – 45.2)	42.9^#^ (40.3 – 44.8)	< 0.05
Albumin < 40 g/L, N (%)	52 (9.0)	63 (10.9)	122 (21.1)^#^	< 0.001
FIB-4	2.12 (1.60 – 2.85)	2.15 (1.58 – 2.89)	2.25^**^ (1.68 – 3.10)	< 0.05
FIB-4 > 2,67	171 (30.4)	173 (30.7)	186 (32.9)	0.61
eGFR CKD-EPI, (mL/min/1.73 m^2^)	85.4 ± 14.6	80.8 ± 16.8^#^	76.0 ± 20.8^#^	< 0.001
eGFR < 45 mL/min/1.73 m^2^, N (%)	6 (1.0)	27 (4.7)^#^	53 (9.1)^#^	< 0.001
Vitamin D (ng/mL)	19.0 (14.5 – 26.4)	18.7 (13.6 – 24.8)	16.3^#^ (12.1 – 22.0)	< 0.001
Vitamin D < 20 ng/ml, N (%)	316 (54.6)	320 (55.3)	391 (67.5)^#^	< 0.001
Survival, N (%)	494 (85.3)	452 (78.1)^**^	399 (68.9)^#^	< 0.001
HR (± 95% CI)	Ref.	1.56^**^ (1.18 – 2.05)	2.32^#^ (1.79 – 3.00)	–
Survival time Q_3_ (years)	Not reached	Not reached	2.8	–
Incidence rate per 1000 (± 95% CI)	26.4 (20.3 – 34.3)	32.3 (25.4 – 41.1)	76.4 (65.1 – 89.8)	–

Data were expressed as mean ± SD or median (interquartile range). Statistics for categorical variables are number of patients (percentage of patients). * p < 0.05; ** p < 0.01; # p < 0.001.

Abbreviations: FGF21, fibroblast growth factor 21; BMI, body mass index; CKD-EPI, Chronic Kidney Disease Epidemiology Collaboration equation; eGFR, estimated glomerular filtration rate; FIB-4, fibrosis 4 index; HbA1c, hemoglobin A1c; HOMA-IR, homeostatic model assessment – insulin resistance; HR, hazard ratio; hs-CRP, high-sensitivity C-reactive protein.

**Table 2:** Baseline clinical characteristics of the female participants according to FGF-21 categories (N = 1775; 50.5% of the analyzed cohort).

	**FGF-21 categories**			
	**LOW [N = 592]**	**MIDDLE [N = 592]**	**HIGH [N = 591]**	***p-value***
FGF-21 (pg/mL)	127.3 (84.7 – 163.4)	288.4 (243.7 – 345.8)	637.8 (497.7 – 947.8)	–
Age (years)	72 ± 8	74 ± 9^**^	77 ± 10^#^	< 0.001
Age ≥ 80 years, N (%)	130 (22.0)	163 (27.5)^*^	260 (44.0)^#^	< 0.001
BMI (kg/m^2^)	28.1 ± 5.3	29.2 ± 5.3^**^	29.6 ± 5.8^#^	< 0.001
Obesity, N (%)	193 (32.9)	247 (42.4)^#^	250 (43.8)^#^	< 0.001
Visceral obesity, N (%)	513 (88.0)	528 (92.1)	527 (92.8)	< 0.01
Arterial hypertension, N (%)	428 (72.3)	448 (75.8)	489 (82.9)^#^	< 0.001
Coronary artery disease, N (%)	42 (34.4)	39 (32.0)	63 (42.6)	0.16
Past stroke, N (%)	26 (4.4)	47 (8.0)^*^	59 (10.0)^#^	< 0.01
Heart failure, N (%)	68 (11.8)	87 (15.3)	111 (20.4)^#^	< 0.001
Diabetes mellitus, N (%)	99 (16.7)	115 (19.4)	189 (32.0)^#^	< 0.001
Impaired fasting glucose, N (%)	160 (27.0)	215 (36.3)^#^	279 (47.2)^#^	< 0.001
Fasting glucose (mg/dL)	93 (87 – 101)	95 (88 – 105)^**^	98 (88 – 112)^#^	< 0.001
HbA1c (mmol/l)	36 (33 – 40)	37 (34 – 41)	38 (34 – 42)^#^	< 0.001
HOMA-IR	1.69 (1.11 – 2.69)	2.00^#^ (1.30 – 3.7)	2.08^#^ (1.23 – 3.31)	< 0.001
HOMA-IR ≥ 2.0	228 (38.7)	295 (50.0)^#^	305 (51.9)^#^	< 0.001
Hypercholesterolemia, N (%)	493 (83.3)	486 (82.1)	447 (75.6)^**^	< 0.01
Total cholesterol (mg/dL)	199.8 ± 44.4	199.8 ± 50.4	189.0 ± 45.7^#^	< 0.001
HDL cholesterol (mg/dL)	60.9 ± 14.8	58.1 ± 14.5^**^	52.4 ± 12.8^#^	< 0.001
LDL cholesterol (mg/dL)	123.2 ± 42.3	126.0 ± 47.1	118.8 ± 43.0	< 0.05
Hypertriglyceridemia, N (%)	110 (18.6)	173 (29.2)^#^	230 (38.9)^#^	< 0.001
Triglycerides (mg/dL)	110 (87 – 139)	122 (100 – 155)^#^	131 (101 – 176)^#^	< 0.001
hs-CRP (mg/L)	1.78 (0.92 – 3.30)	2.11^**^ (1.15 – 4.35)	2.98^#^ (1.34 – 6.01)	< 0.001
hs-CRP > 3 mg/L, N (%)	164 (27.7)	208 (35.1)^**^	295 (49.9)^#^	< 0.001
hs-CRP > 5 mg/L, N (%)	85 (14.4)	123 (20.8)^**^	185 (31.3)^#^	< 0.001
Hyperuricemia, N (%)	94 (15.9)	180 (30.4)^#^	241 (40.8)^#^	< 0.001
Uric acid (mg/dl)	4.98 ± 1.26	5.47 ± 1.45^#^	5.87 ± 1.74^#^	< 0.001
Statins, N (%)	207 (35.0)	219 (37.0)	212 (35.9)	0.77
Fibrates, N (%)	4 (0.7)	3 (0.5)	26 (4.4)^#^	< 0.001
Albumin (g/L)	43.5 (41.8 – 45.2)	43.6 (41.8 – 45.0)	42.6^#^ (40.8 – 44.5)	< 0.001
Albumin < 40 g/L, N (%)	48 (8.1)	61 (10.3)	115 (15.5)^#^	< 0.001
FIB-4	1.84 (1.44 – 2.43)	1.88 (1.46 – 2.55)	1.99^**^ (1.52 – 2.65)	< 0.01
FIB-4 > 2,67	102 (17.8)	121 (21.1)	140 (24.6)^**^	< 0.05
eGFR CKD-EPI, (mL/min/1.73 m^2^)	85.2 ± 14.7	80.9 ± 17.1^#^	72.0 ± 22.0^#^	< 0.001
eGFR < 45 mL/min/1.73 m^2^, N (%)	12 (2.0)	26 (4.4)	85 (14.4)^#^	< 0.001
Vitamin D (ng/mL)	20.2 (14.2 – 27.5)	18.0^*^ (13.5 – 25.2)	16.8^#^ (11.5 – 24.7)	< 0.001
Vitamin D < 20 ng/mL, N (%)	292 (49.4)	348 (58.8)^**^	370 (62.6)^#^	< 0.001
Survival, N (%)	535 (90.4)	525 (88.7)	443 (75.0)^#^	< 0.001
HR (± 95% CI)	Ref.	1.23 (0.86 – 1.75)	2.93^#^ (2.15 – 3.98)	–
Survival time Q_3_ (years)	Not reached	Not reached	3.9	–
Incidence rate per 1000 (± 95% CI)	42.5 (34.4 – 52.6)	66.1 (55.6 – 78.7)	98.0 (84.7 – 113.4)	–

Data were expressed as mean ± SD or median (interquartile range). Statistics for categorical variables are number of patients (percentage of patients). * p < 0.05; ** p < 0.01; # p < 0.001.

Abbreviations: FGF21, fibroblast growth factor 21; BMI, body mass index; CKD-EPI, Chronic Kidney Disease Epidemiology Collaboration equation; eGFR, estimated glomerular filtration rate; FIB-4, fibrosis 4 index; HbA1c, hemoglobin A1c; HOMA-IR, homeostatic model assessment – insulin resistance; HR, hazard ratio; hs-CRP, high-sensitivity C-reactive protein.

During a follow-up of 4.19 years, the survival rates for low, middle, and high FGF21 tertiles were 85.3%, 78.1%, and 68.9% for men; and 90.4%, 88.7%, and 75.0% for women, respectively.

### Outcome data

**Figure 1:**
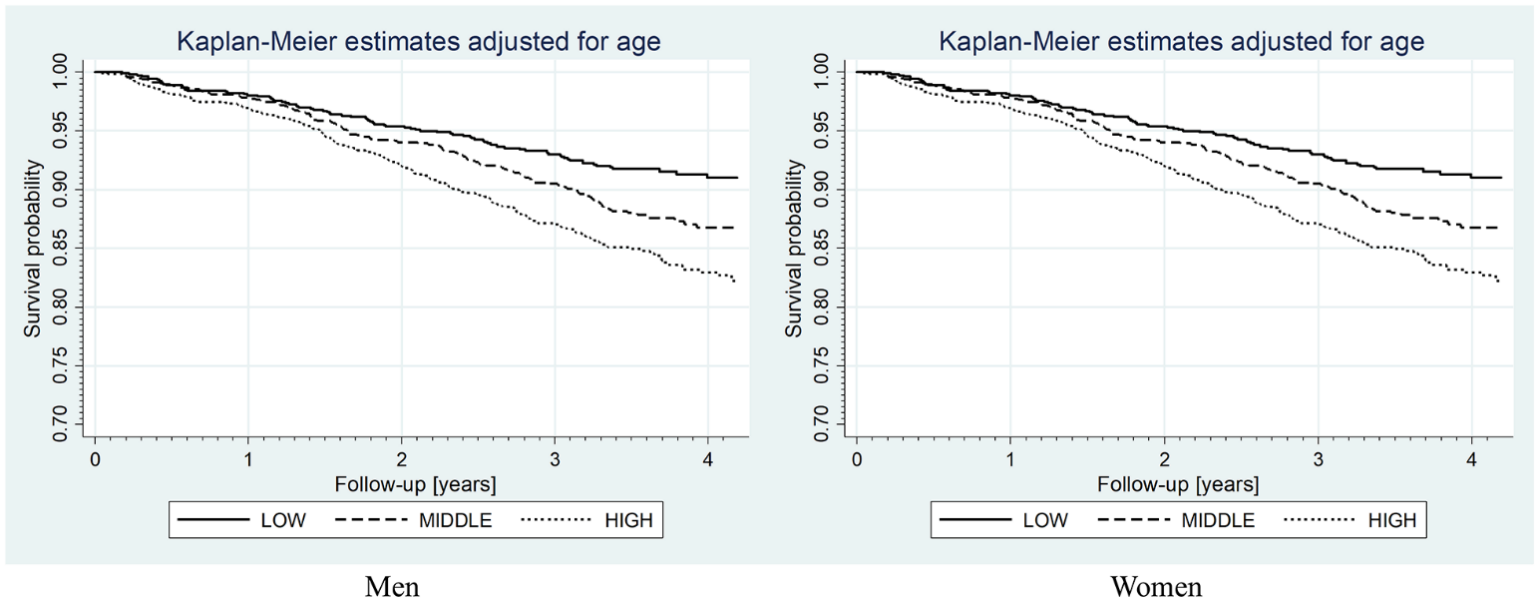
**Kaplan-Meier curves for overall survival rates according to FGF-21 level categories.** Log-rank test: for men, low vs. middle *p* < 0.01, low vs. high *p* < 0.001, middle vs. high *p* < 0.01; for women, low vs. middle *p* = 0.7, low vs. high *p* < 0.001, middle vs. high *p* < 0.001.

Kaplan-Meier analysis indicated that both in men and women cumulative survival rate was worse in participants with high vs. low serum FGF21 levels (log-rank test, p < 0.001) and high vs. middle FGF21 levels (log-rank test, p < 0.01 for men and p < 0.001 for women) – Figure 1. In the univariable Cox regression analysis (crude and age-adjusted), factors decreasing survival enclose: higher FGF21 levels, older age, diabetes, heart failure, past stroke, impaired kidney function, inflammatory state, vitamin D deficiency, lower albumin level, increased FIB-4 (fibrosis 4 index) values, and hyperuricemia (Table 3). On the other hand, female sex, visceral obesity and hypercholesterolemia were protective factors. Obesity, hypertriglyceridemia and insulin resistance lost their protective effect in age-adjusted analysis.

**Table 3:** The results of univariate and multivariate Cox regression analyses for all-cause mortality.

	**Univariable**		**Multivariable**
	**HR (± 95% CI)**	**HR (± 95% CI) age-adjusted**	**HR (± 95% CI)**
FGF-21 [pg/mL] per 1-SD	1.34^#^ (1.28 – 1.41)	1.24^#^ (1.17 – 1.30)	
• FGF-21 LOW	Ref.	Ref.	Ref.
• FGF-21 MIDDLE	1.43^**^ (1.14 – 1.77)	1.31^*^ (1.05 – 1.62)	1.23 (0.97 – 1.56)
• FGF-21 HIGH	2.56^#^ (2.10 – 3.12)	1.94^#^ (1.59 – 2.37)	1.53^#^ (1.22 – 1.92)
Female	0.65^#^ (0.55 – 0.75)	0.65^#^ (0.56 – 0.76)	0.75^**^ (0.63 – 0.90)
Age ≥ 80 years	5.57^#^ (4.73 – 6.56)	–	3.57^#^ (2.93- 4.36)
Obesity	0.73^#^ (0.61 – 0.87)	0.88 (0.74 – 1.04)	
Visceral obesity	0.56^#^ (0.47 – 0.69)	0.61^#^ (0.51 – 0.74)	0.61^#^ (0.49 – 0.75)
Arterial hypertension	1.09 (0.91 – 1.32)	0.88 (0.74 – 1.07)	
Past stroke	2.17^#^ (1.76 – 2.67)	1.70^#^ (1.38 – 2.10)	1.50^**^ (1.19 – 1.90)
Heart failure	2.04^#^ (1.72 – 2.42)	1.39^#^ (1.16 – 1.66)	1.27^*^ (1.05 – 1.53)
eGFR < 45 mL/min/1.73 m^2^	3.94^#^ (3.20 – 4.85)	1.99^#^ (1.60 – 2.46)	1.61^#^ (1.25 – 2.07)
Diabetes mellitus	1.52^#^ (1.29 – 1.79)	1.48^#^ (1.26 – 1.74)	1.46^#^ (1.21 – 1.75)
Impaired fasting glucose	1.06 (0.91 – 1.23)	1.15 (0.98 – 1.34)	
HOMA-IR ≥ 2.0	0.83^*^ (0.71 – 0.97)	0.95 (0.82 – 1.11)	
Hypercholesterolemia	0.51^#^ (0.44 – 0.61)	0.61^#^ (0.52 – 0.71)	0.74^**^ (0.62 – 0.89)
Statins	0.96 (0.82 – 1.12)	0.89 (0.76 – 1.04)	
Hypertriglyceridemia	0.68^#^ (0.56 – 0.82)	0.84 (0.69 – 1.01)	
Hyperuricemia	1.69# (1.45 – 1.98)	1.45^#^ (1.24 – 1.69)	1.26^*^ (1.05 – 1.50)
hs-CRP > 3 mg/L	2.00^#^ (1.72 – 2.33)	1.91^#^ (1.64 – 2.23)	1.64^#^ (1.37 – 1.95)
Albumin < 40 g/L	3.46^#^ (2.93 – 4.09)	2.07^#^ (1.74 – 2.46)	1.39^**^ (1.13 – 1.71)
FIB4 > 2.67	2.33^#^ (2.00 – 2.73)	1.30^**^ (1.10 – 1.54)	1.28^**^ (1.07 – 1.53)
Vitamin D < 20 ng/mL	1.74^#^ (1.78 – 2.06)	1.36^#^ (1.15 – 1.61)	1.26^*^ (1.04 – 1.51)

*p < 0.05; ** p < 0.01; # p < 0.001.

Abbreviations: FGF21, fibroblast growth factor 21; eGFR, estimated glomerular filtration rate; FIB-4, fibrosis 4 index; HOMA-IR, homeostatic model assessment – insulin resistance; HR, hazard ratio; hs-CRP, high-sensitivity C-reactive protein.

All factors significant in univariable analysis were included in the multivariable analysis, yielding risk factors such as: the highest FGF21 group, older age, past stroke, diabetes, heart failure, impaired kidney function, inflammatory state, vitamin D deficiency, lower albumin level, FIB-4 values over 2.67 and hyperuricemia. Moreover, protective factors were female sex, visceral obesity and hypercholesterolemia (Table 3).

So, the analysis mentioned above, after adjustment for the confounding factors, indicated that a high serum level of FGF21 is an independent predictor of all-cause mortality (HR 1.53; 95% CI: 1.22 – 1.92).

**Figure 2:**
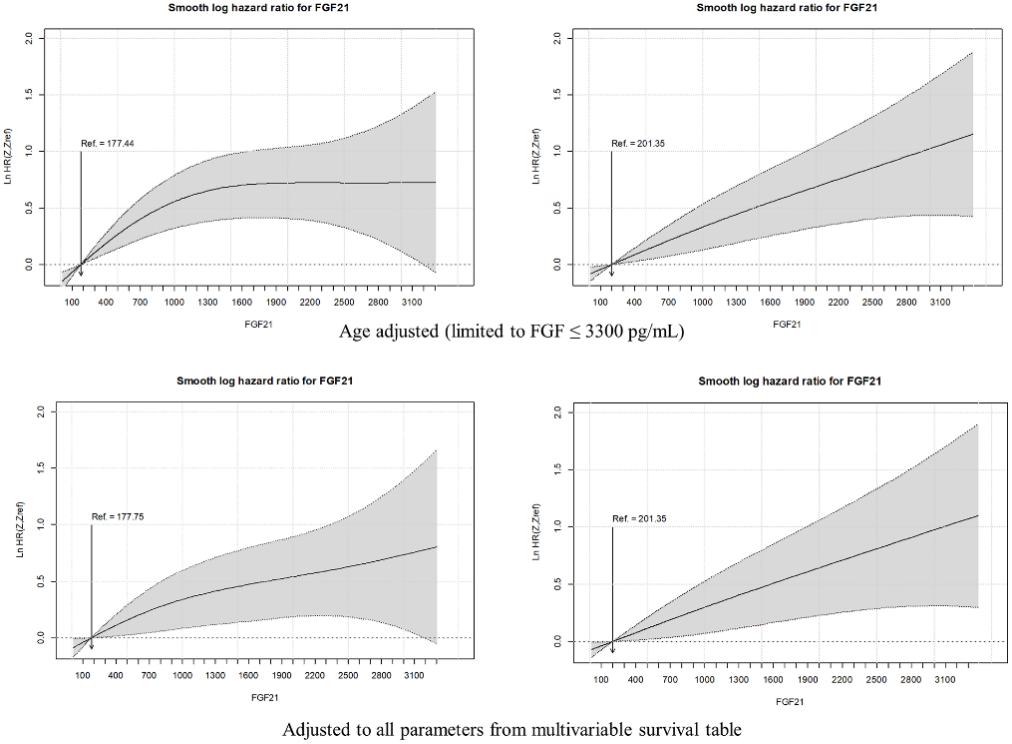
**Smooth hazard ratios for FGF2l levels in men and women, adjusted to age and all significant factors from multivariable Cox regression analysis.** The gray area indicates the 95 % confidence interval.

Smooth hazard ratios for FGF21 levels in men and women, adjusted to age and all significant factors from multivariable Cox regression analysis are presented in Figure 2. One may see that for men the age-adjusted curve (from the cut-off point equal to sex-specific FGF21 tercile) rises to approximately 1500 pg/mL and further flattens, while for women increases steadily. In the case of multivariable analysis for both genders, the risk of death increases steadily, yet to the lower values for men in comparison to women.

## DISCUSSION

The current study was part of a large-scale, multi-centre prospective project indicating that FGF21 serum concentration may play a role as a predictor of all-cause mortality in the general population of older individuals. In our study, we found a significant inverse correlation between circulating FGF21 and survival rate, which was significantly lower in the middle and high-level FGF21 groups compared to the low-level FGF21 subgroup. Of note, participants with high serum levels of FGF21 more frequently had metabolic complications, such as hypertension, obesity, diabetes, hypercholesterolemia, and hypertriglyceridemia. Serum FGF21 level increases with age, which was particularly profound in subjects above 80 years of age.

Our findings are in line with a previous multicenter Italian study on a large group of patients aged from 21 to 113 years, in whom serum FGF21 level was positively correlated with age and inversely correlated with survival in nonagenarians and centenarians [10]. It was also associated with aggravated functional and biochemical parameters (lower handgrip strength, higher BMI, insulin resistance, and increased CRP, creatinine, triglycerides, and uric acid levels), particularly in the eldest. In another recent study [11] elevated serum FGF21 concentration was an independent predictor of all-cause mortality, major adverse cardiovascular events, and pneumonia in hemodialysis patients. Similar findings provided studies on patients with acute heart failure [12], diabetes and coronary artery calcifications, and HIV/HCV coinfection [13]. However, studies on the relationship between FGF21 and longevity in humans are inconsistent. A prospective cohort study of 1668 patients with coronary artery disease yielded a U-shaped correlation between serum FGF21 levels and mortality, with both higher and lower serum FGF21 levels associated with increased cardiovascular as well as all-cause mortality [14].

The available literature has indicated so far, a potential beneficial effect of increased FGF21 on lifespan in experimental models [6]. The anti-aging properties could be attributed to a wide range of mechanisms influencing metabolism, such as the regulation of glucose and lipid metabolism, induction of adiponectin secretion from fat tissue, a corticotropin-releasing hormone from the hypothalamus [15], and GLUT1 (glucose transporter 1) expression, which leads to improvement of autophagy in targeted tissues, hypothalamic-pituitary-adrenal axis regulation and secretion of the increase of glucose uptake [15, 16]. These are just a few of the many postulated anti-aging properties of FGF21.

One of the most important factors regulating hepatic production of FGF21, both in rodents and humans, is fasting, particularly a protein-restriction diet. This specific stimulus increases the hepatic production of FGF21 via peroxisome proliferator-activated receptor alpha (PPARα) and does not affect it in other tissues or may even decrease its expression in the pancreas [17, 18]. Interestingly, two entirely different stimuli, overfeeding and obesity both enhance FGF21 production, predominantly in the pancreas and white adipose tissue probably affecting its liver secretion to a lesser extent [19]. Both fasting and overfeeding represent metabolic stressors that disrupt homeostasis, triggering increase of FGF21 levels to adapt to these states. This is just an exemplification of the complex interplay between numerous exogenous factors influencing FGF21 secretion.

In turn, gender is among endogenous factors affecting substantially FGF21 concentration. Sex and reproductive status are essential biological variables influencing FGF21-induced weight loss, with favorable effect in male subjects. Higher levels of FGF21 observed in our study in female patients are in line with previous findings [20–22]. Nevertheless, the difference in FGF21 levels between males and females cannot be solely attributed to hormonal divergence, since women included in our study were postmenopausal. Experimental studies indicate that not all metabolic endpoints of FGF21 administration can be attributed to estrogen levels. The decrease of hepatic steatosis under the influence of FGF21 is less effective in female rodents, but it is not conditioned by ovarian hormone status. Another study has shown that estrogen treatment restores serum FGF21 concentrations and reduces NAFLD in female rats probably via activation of hepatic estrogen receptor α [23]. However, these findings were not reproduced in an observational cohort of menopausal women who received E2 therapy. The implication of gender differences in FGF21 levels remains an interesting area for future research, especially in the context of higher FGF21 concentrations and survival rates in female patients presented in this study.

One of the issues disrupting the interpretation of FGF21 regulation is varying responses to some stimuli on FGF21 secretion in different species, i.e. fasting increases the serum level of FGF21 within hours in mice, while in humans after 3-7 days. Furthermore, mice fed with a ketogenic diet have increased levels of FGF21, which was not observed in humans [17]. Another discrepancy between the two experimental models is the different pathways of FGF21 degradation. Unlike in mice, in humans, it is inactivated by endopeptidase fibroblast activation protein [24] – which is currently considered a potential therapeutic target to increase endogenous FGF21 activity. Mentioned above ambiguities bring into question whether experimental conditions should be directly transferred to the human model.

Experimental studies have shown that FGF21 level may decrease or increase with age depending on the model [25, 26]. Transgenic mice overexpressing FGF21 had higher concentrations of FGF21, which was associated with an extended lifespan, reaching a 36% increase in the median survival time, even more evident, in transgenic females [6]. In addition, transgenic FGF21 mice had decreased levels of insulin and insulin-like growth factor-1 (IGF1), glucose, and triglycerides, while elevated growth hormone and adiponectin levels. This finding indicated FGF21 involvement in re-establishing metabolism and hormonal balance towards more preferable ones. The increase in lifespan may be partially due to a decrease in IGF1 as a consequence of GH resistance observed in FGF21 transgenic mice [7]. Also *in vitro* studies imply the pro-longevity properties of FGF21. Co-cultivation of human umbilical vascular endothelial cells with FGF21 delayed cell senescence as evidenced by increased levels of proteins P53 and P21 and restored levels of sirtuin 1–anti-aging protein playing an essential role in atherosclerosis vascular aging prevention [27].

In humans, in physiological conditions, circulating FGF21 concentrations increase with age [9]. FGF21 serum levels are also increased in metabolic conditions such as obesity, type 2 diabetes and cardiovascular disease. These findings are consistent with our results. While previous studies have established a link between age and FGF21 levels in the general population and in specific groups of patients (with cardiovascular disease and end-stage kidney disease [10–12]), our study uniquely explores the relationship between FGF21 levels and health status in a large group of individuals aged 60 years and older. Our study shows that FGF21 increase is particularly expressed in obesity and obesity-related conditions, and it is an independent predictor of all-cause mortality.

Taking into account that healthy human aging is associated with increased FGF21 level, further elevation of its concentration above physiological level in subjects with obesity, diabetes, chronic kidney disease, and cardiovascular disease may be considered as a compensation/counteraction mechanism of pathological metabolic changes or, it may be just a reflection of multi-organ failure. On the other hand, it may also be a consequence of FGF21 resistance, for example, due to reduced expression of the FGF21 receptor complex [1, 28]. These theories have not been well established, but regardless of the pathomechanisms behind these changes in serum concentration, FGF21 may be considered a useful predictive biomarker of clinical outcomes and mortality or to put it in other terms, a parameter indicative of the health status.

There are already known non-pharmacological interventions that indirectly decrease FGF21 concentration such as physical activity and fasting [29]. On the contrary, available data from clinical trials indicating beneficial effect of FGF21 analogues in managing weight loss, hyperinsulinemia and dyslipidemia are promising but of insufficient quality [30]. Their effect on mortality remains also inconclusive. This raises the question whether antiobesity effects of FGF21 analogues observed in rodents can be translated to humans.

Understanding the relationship between increased FGF21 concentration and increased mortality will help develop effective FGF21-based pharmacotherapy targeting obesity, diabetes and other metabolic disorders. Referring to this, it is reasonable to continue large-scale studies for establishing a range of sex- and age-dependent reference values for serum FGF21 based on general population analyses, which in turn may help to assess individual responsiveness to the treatment with FGF21 analogues.

## MATERIALS AND METHODS

### Study design

**Figure 3:**
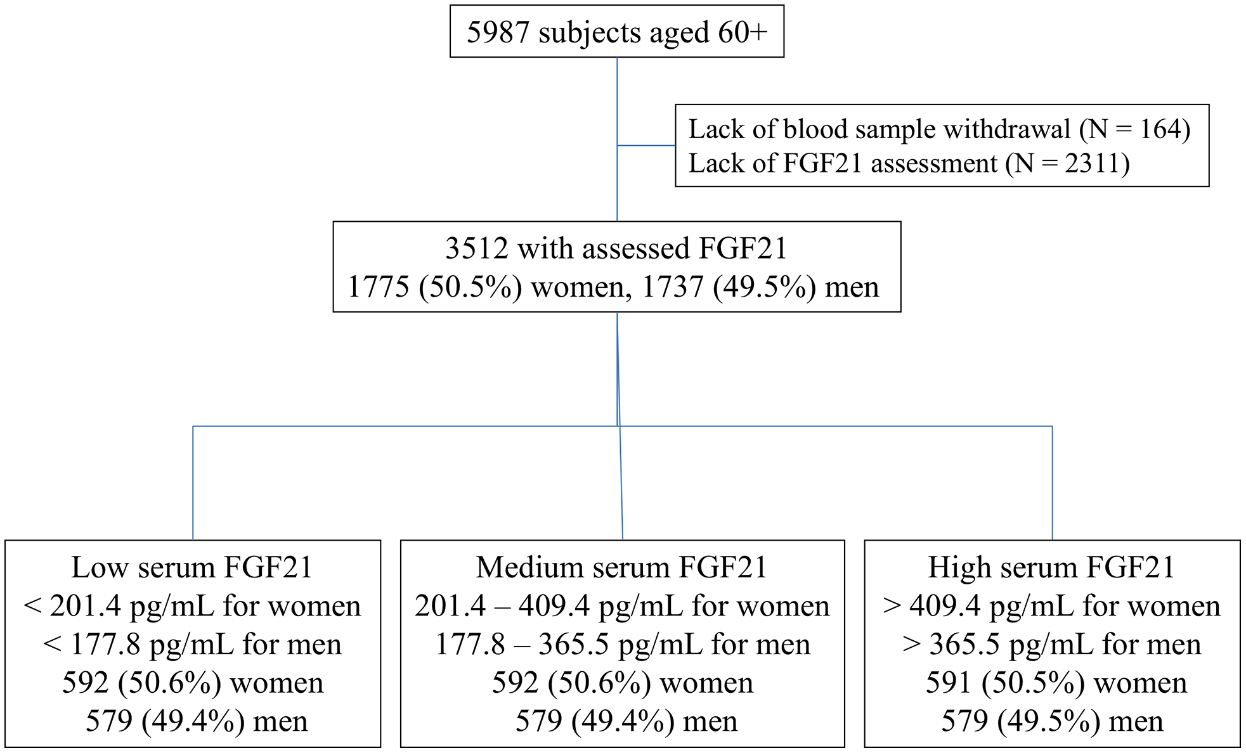
Flow chart.

The current analysis was a part of the PolSenior2 study – a large, multicentre, interdisciplinary project conducted between January 2018 and December 2019. A multistage, stratified and clustered sampling design was used for subject recruitment in order to obtain a sample representative for old and very old citizens in Poland, with 99% of them being Caucasians. The study procedures included structured surveys, geriatric tests and scales, along with anthropometric and blood pressure measurements performed during three home visits. As described in the flow-chart (Figure 3), 5987 individuals aged 60 and older were recruited and divided into seven age groups of equal size and gender distribution (60-65, 65-69, 70-74, 75-79, 80-84, 85-89 and ≥90 years) according to the protocol previously described in detail elsewhere [31] and online at https://polsenior2.mug.edu.pl/. Such study design was aimed at increasing the statistical power of analyzes performed in the oldest age groups. As shown on the analysis flow chart, 3512 participants were included in the final analysis. Due to the lack of blood samples (N = 164) or lack of FGF21 assessment (which was not initially planned for the purpose of the study and at the stage of measurements performed for the present sub-study a large number of samples were not available in the biobank; N = 2311), a total number of 2475 individuals were not included.

The analysis was based on the structured questionnaires, which included present health status, history of diseases and clinical conditions (obesity, diabetes, impaired fasting glucose, hypertension, coronary artery disease, past stroke, hospitalization for heart failure, hyperuricemia), current medication use, and socioeconomic data. The results were reported and the manuscript was prepared according to STROBE guidelines.

### Data analysis

For this study subjects were categorized according to serum FGF21 concentrations into 3 equally sized subgroups (tertiles) for each sex separately. Survival data were acquired from the Polish population register (PESEL). At the moment of checking the register, 664 individuals had died (392 men and 272 women) and the total number of person-years of observation was 11884.8 with an incidence rate of 55.8 (95% CI: 51.7 – 60.2) per 1000 subjects.

Serum samples were collected from the participants and had been stored at -80° C until analysis. Laboratory assessments were performed in a laboratory dedicated to the PolSenior2 project (Bruss Laboratory in Gdynia). Blood sample analysis of total cholesterol, LDL and HDL fractions, triglycerides, hs-CRP (high-sensitivity C-reactive protein), 25-OH vitamin D, albumin, uric acid, insulin, glucose, HbA1c (haemoglobin A1c), and creatinine was assessed using Atellica Solution Immunoassay and Clinical Chemistry Analyzers and reagents from Siemens Healthineers. Inter-assay coefficients of variability for serum total cholesterol, LDL, HDL, triglycerides, uric acid, glucose, creatinine (enzymatic method): 2.6%, 4.5%, 3%, 2.2%, 3.3%, 4.9%, 1.2%, respectively; for serum CRP (hsCRP) concentration (immunoturbidimetric method): 4%; for serum insulin and 25-OH vitamin D (immunochemiluminescent method): 3.8% and 10.8%, respectively; for albumin (spectrophotometric method): 1.6%; for HbA1c (high-performance liquid chromatography): 1.5%.

Serum FGF21 concentration was measured by BioVendor (Modrice, Czech Republic) in the Laboratory of the Department of Pathophysiology in Katowice, with the lower limit of detection of 7 pg/mL and intra- and inter-assay coefficients of variability were 3.5% and 3.7%.

### Statistical analysis

Statistical analyses were performed using STATISTICA 13.0 PL (TIBCO Software Inc., CA, USA), and Stata SE 13.0 (StataCorp LP, TX, USA). Statistical significance was set at a p-value below 0.05. All tests were two-tailed. Imputations were not performed for missing data. Nominal and ordinal data were expressed as percentages. Interval data were expressed as the mean value ± standard deviation in the case of normal distribution. In the case of data with skewed or non-normal distribution, they were expressed as the median, with lower and upper quartiles. The distribution of variables was evaluated by the Anderson-Darling test and the quantile–quantile (Q–Q) plot. The homogeneity of variances was assessed by the Levene test. Nominal and ordinal data were compared with the χ2 test. Comparisons between groups for interval data were done with a one-way analysis of variances with Dunnett’s post-hoc test. Overall mortality risk factors were assessed with a Pointwise Nonparametric Estimation of Hazard Ratio method (R package smoothHR). The FGF21 were modeled as p-splines with automatically fitted degrees of freedom for flexible continuous covariates in multivariate additive Cox models. Results were presented with hazard ratios and graphically with smooth HR plots. The proportionality assumption was tested based on the Schoenfeld residuals (R function cox.zph). Multiple-collinearity was checked based on the correlation matrix of coefficients of the survival model. Additionally, overall survival analyses were done with Kaplan-Meyer curves, stratified by sex, with the log-rank test to compare survival curves.

## References

[1] Chen Z, Yang L, Liu Y, Huang P, Song H, Zheng P. The potential function and clinical application of FGF21 in metabolic diseases. Front Pharmacol. 2022; 13:1089214. 10.3389/fphar.2022.1089214 36618930 PMC9810635

[2] Yang C, Wang C, Ye M, Jin C, He W, Wang F, McKeehan WL, Luo Y. Control of lipid metabolism by adipocyte FGFR1-mediated adipohepatic communication during hepatic stress. Nutr Metab (Lond). 2012; 9:94. 10.1186/1743-7075-9-94 23106963 PMC3545967

[3] Foltz IN, Hu S, King C, Wu X, Yang C, Wang W, Weiszmann J, Stevens J, Chen JS, Nuanmanee N, Gupte J, Komorowski R, Sekirov L, et al. Treating diabetes and obesity with an FGF21-mimetic antibody activating the βKlotho/FGFR1c receptor complex. Sci Transl Med. 2012; 4:162ra153. 10.1126/scitranslmed.3004690 23197570

[4] Talukdar S, Zhou Y, Li D, Rossulek M, Dong J, Somayaji V, Weng Y, Clark R, Lanba A, Owen BM, Brenner MB, Trimmer JK, Gropp KE, et al. A Long-Acting FGF21 Molecule, PF-05231023, Decreases Body Weight and Improves Lipid Profile in Non-human Primates and Type 2 Diabetic Subjects. Cell Metab. 2016; 23:427–40. 10.1016/j.cmet.2016.02.001 26959184

[5] Tan H, Yue T, Chen Z, Wu W, Xu S, Weng J. Targeting FGF21 in cardiovascular and metabolic diseases: from mechanism to medicine. Int J Biol Sci. 2023; 19:66–88. 10.7150/ijbs.73936 36594101 PMC9760446

[6] Zhang Y, Xie Y, Berglund ED, Coate KC, He TT, Katafuchi T, Xiao G, Potthoff MJ, Wei W, Wan Y, Yu RT, Evans RM, Kliewer SA, Mangelsdorf DJ. The starvation hormone, fibroblast growth factor-21, extends lifespan in mice. Elife. 2012; 1:e00065. 10.7554/eLife.00065 23066506 PMC3466591

[7] Salminen A, Kaarniranta K, Kauppinen A. Regulation of longevity by FGF21: Interaction between energy metabolism and stress responses. Ageing Res Rev. 2017; 37:79–93. 10.1016/j.arr.2017.05.004 28552719

[8] Tezze C, Romanello V, Desbats MA, Fadini GP, Albiero M, Favaro G, Ciciliot S, Soriano ME, Morbidoni V, Cerqua C, Loefler S, Kern H, Franceschi C, et al. Age-Associated Loss of OPA1 in Muscle Impacts Muscle Mass, Metabolic Homeostasis, Systemic Inflammation, and Epithelial Senescence. Cell Metab. 2017; 25:1374–89.e6. 10.1016/j.cmet.2017.04.021 28552492 PMC5462533

[9] Hanks LJ, Gutiérrez OM, Bamman MM, Ashraf A, McCormick KL, Casazza K. Circulating levels of fibroblast growth factor-21 increase with age independently of body composition indices among healthy individuals. J Clin Transl Endocrinol. 2015; 2:77–82. 10.1016/j.jcte.2015.02.001 26042208 PMC4450097

[10] Conte M, Ostan R, Fabbri C, Santoro A, Guidarelli G, Vitale G, Mari D, Sevini F, Capri M, Sandri M, Monti D, Franceschi C, Salvioli S. Human Aging and Longevity Are Characterized by High Levels of Mitokines. J Gerontol A Biol Sci Med Sci. 2019; 74:600–7. 10.1093/gerona/gly153 29955888

[11] Li M, Jiang LQ, Zhang MY, Liu SS, Sawh RR, Zheng J, Yan Y, Hou SM, Lu KQ, Thorne O, Liu BC, Qian Q, Wu YF, et al. Elevated serum FGF21 is an independent predictor for adverse events in hemodialysis patients from two large centers: a prospective cohort study. Ren Fail. 2023; 45:2256414. 10.1080/0886022X.2023.2256414 37724523 PMC10512844

[12] Shen Y, Zhang X, Pan X, Xu Y, Xiong Q, Lu Z, Ma X, Bao Y, Jia W. Contribution of serum FGF21 level to the identification of left ventricular systolic dysfunction and cardiac death. Cardiovasc Diabetol. 2017; 16:106. 10.1186/s12933-017-0588-5 28821258 PMC5562996

[13] Shengir M, Fillebeen C, Wagner J, Ramanakumar AV, Kaouache M, Klein MB, Pantopoulos K, Sebastiani G. Increased Serum Fibroblast Growth Factor 23 Predicts Mortality in People With HIV/HCV Coinfection. J Acquir Immune Defic Syndr. 2023; 94:273–9. 10.1097/QAI.0000000000003245 37368933

[14] Li Q, Zhang Y, Ding D, Yang Y, Chen Q, Su D, Chen X, Yang W, Qiu J, Ling W. Association Between Serum Fibroblast Growth Factor 21 and Mortality Among Patients With Coronary Artery Disease. J Clin Endocrinol Metab. 2016; 101:4886–94. 10.1210/jc.2016-2308 27662438

[15] Owen BM, Ding X, Morgan DA, Coate KC, Bookout AL, Rahmouni K, Kliewer SA, Mangelsdorf DJ. FGF21 acts centrally to induce sympathetic nerve activity, energy expenditure, and weight loss. Cell Metab. 2014; 20:670–7. 10.1016/j.cmet.2014.07.012 25130400 PMC4192037

[16] Ge X, Chen C, Hui X, Wang Y, Lam KS, Xu A. Fibroblast growth factor 21 induces glucose transporter-1 expression through activation of the serum response factor/Ets-like protein-1 in adipocytes. J Biol Chem. 2011; 286:34533–41. 10.1074/jbc.M111.248591 21846717 PMC3186365

[17] Christodoulides C, Dyson P, Sprecher D, Tsintzas K, Karpe F. Circulating fibroblast growth factor 21 is induced by peroxisome proliferator-activated receptor agonists but not ketosis in man. J Clin Endocrinol Metab. 2009; 94:3594–601. 10.1210/jc.2009-0111 19531592

[18] Spann RA, Morrison CD, den Hartigh LJ. The Nuanced Metabolic Functions of Endogenous FGF21 Depend on the Nature of the Stimulus, Tissue Source, and Experimental Model. Front Endocrinol (Lausanne). 2022; 12:802541. 10.3389/fendo.2021.802541 35046901 PMC8761941

[19] Singhal G, Fisher FM, Chee MJ, Tan TG, El Ouaamari A, Adams AC, Najarian R, Kulkarni RN, Benoist C, Flier JS, Maratos-Flier E. Fibroblast Growth Factor 21 (FGF21) Protects against High Fat Diet Induced Inflammation and Islet Hyperplasia in Pancreas. PLoS One. 2016; 11:e0148252. 10.1371/journal.pone.0148252 26872145 PMC4752212

[20] Chaffin AT, Larson KR, Huang KP, Wu CT, Godoroja N, Fang Y, Jayakrishnan D, Soto Sauza KA, Sims LC, Mohajerani N, Goodson ML, Ryan KK. FGF21 controls hepatic lipid metabolism via sex-dependent interorgan crosstalk. JCI Insight. 2022; 7:e155848. 10.1172/jci.insight.155848 35998055 PMC9675565

[21] Rodgers M, Heineman B, Dushay J. Increased fructose consumption has sex-specific effects on fibroblast growth factor 21 levels in humans. Obes Sci Pract. 2019; 5:503–10. 10.1002/osp4.360 31687174 PMC6819978

[22] Bisgaard A, Sørensen K, Johannsen TH, Helge JW, Andersson AM, Juul A. Significant gender difference in serum levels of fibroblast growth factor 21 in Danish children and adolescents. Int J Pediatr Endocrinol. 2014; 2014:7. 10.1186/1687-9856-2014-7 24883065 PMC4039053

[23] Allard C, Bonnet F, Xu B, Coons L, Albarado D, Hill C, Fagherazzi G, Korach KS, Levin ER, Lefante J, Morrison C, Mauvais-Jarvis F. Activation of hepatic estrogen receptor-α increases energy expenditure by stimulating the production of fibroblast growth factor 21 in female mice. Mol Metab. 2019; 22:62–70. 10.1016/j.molmet.2019.02.002 30797705 PMC6437689

[24] Dunshee DR, Bainbridge TW, Kljavin NM, Zavala-Solorio J, Schroeder AC, Chan R, Corpuz R, Wong M, Zhou W, Deshmukh G, Ly J, Sutherlin DP, Ernst JA, Sonoda J. Fibroblast Activation Protein Cleaves and Inactivates Fibroblast Growth Factor 21. J Biol Chem. 2016; 291:5986–96. 10.1074/jbc.M115.710582 26797127 PMC4786731

[25] Fujita Y, Makishima M, Bhawal UK. Differentiated embryo chondrocyte 1 (DEC1) is a novel negative regulator of hepatic fibroblast growth factor 21 (FGF21) in aging mice. Biochem Biophys Res Commun. 2016; 469:477–82. 10.1016/j.bbrc.2015.12.045 26697751

[26] Villarroya J, Gallego-Escuredo JM, Delgado-Anglés A, Cairó M, Moure R, Gracia Mateo M, Domingo JC, Domingo P, Giralt M, Villarroya F. Aging is associated with increased FGF21 levels but unaltered FGF21 responsiveness in adipose tissue. Aging Cell. 2018; 17:e12822. 10.1111/acel.12822 30043445 PMC6156525

[27] Yan J, Wang J, Huang H, Huang Y, Mi T, Zhang C, Zhang L. Fibroblast growth factor 21 delayed endothelial replicative senescence and protected cells from H_2_O_2_-induced premature senescence through SIRT1. Am J Transl Res. 2017; 9:4492–501. 29118911 PMC5666058

[28] Larsson SC, Michaëlsson K, Mola-Caminal M, Höijer J, Mantzoros CS. Genome-wide association and Mendelian randomization study of fibroblast growth factor 21 reveals causal associations with hyperlipidemia and possibly NASH. Metabolism. 2022; 137:155329. 10.1016/j.metabol.2022.155329 36208799

[29] Cuevas-Ramos D, Almeda-Valdés P, Meza-Arana CE, Brito-Córdova G, Gómez-Pérez FJ, Mehta R, Oseguera-Moguel J, Aguilar-Salinas CA. Exercise increases serum fibroblast growth factor 21 (FGF21) levels. PLoS One. 2012; 7:e38022. 10.1371/journal.pone.0038022 22701542 PMC3365112

[30] Carbonetti MP, Almeida-Oliveira F, Majerowicz D. Use of FGF21 analogs for the treatment of metabolic disorders: a systematic review and meta-analysis. Arch Endocrinol Metab. 2023; 68:e220493. 10.20945/2359-4292-2022-0493 37948566 PMC10916804

[31] Wierucki Ł, Kujawska-Danecka H, Mossakowska M, Grodzicki T, Błędowski P, Chudek J, Kostka T, Więcek A, Hajduk A, Bandosz P, Zagożdżon P, Wojtyniak B, Zdrojewski T. Health status and its socio-economic covariates in the older population in Poland - the assumptions and methods of the nationwide, cross-sectional PolSenior2 survey. Arch Med Sci. 2020; 18:92–102. 10.5114/aoms.2020.100898 35154530 PMC8826695

